# Predictive Role of Executive Function in the Efficacy of Intermittent Theta Burst Transcranial Magnetic Stimulation Modalities for Treating Methamphetamine Use Disorder—A Randomized Clinical Trial

**DOI:** 10.3389/fpsyt.2021.774192

**Published:** 2021-12-02

**Authors:** Li-Jin Wang, Lin-Lin Mu, Zi-Xuan Ren, Hua-Jun Tang, Ya-Dong Wei, Wen-Juan Wang, Pei-Pei Song, Lin Zhu, Qiang Ling, He Gao, Lei Zhang, Xun Song, Hua-Feng Wei, Lei-Xin Chang, Tao Wei, Yu-Jing Wang, Wei Zhao, Yan Wang, Lu-Ying Liu, Yi-Ding Zhou, Rui-Dong Zhou, Hua-Shan Xu, Dong-Liang Jiao

**Affiliations:** ^1^School of Mental Health, Bengbu Medical College, Bengbu, China; ^2^Compulsory Isolated Drug Rehabilitation Center, Bengbu, China

**Keywords:** methamphetamine, repetitive transcranial magnetic stimulation, intermittent theta burst stimulation, executive function, craving

## Abstract

**Background:** Repetitive transcranial magnetic stimulation (rTMS) has therapeutic effects on craving in methamphetamine (METH) use disorder (MUD). The chronic abuse of METH causes impairments in executive function, and improving executive function reduces relapse and improves treatment outcomes for drug use disorder. The purpose of this study was to determine whether executive function helped predict patients' responses to rTMS treatment.

**Methods:** This study employed intermittent theta burst stimulation (iTBS) rTMS modalities and observed their therapeutic effects on executive function and craving in MUD patients. MUD patients from an isolated Drug Rehabilitation Institute in China were chosen and randomly allocated to the iTBS group and sham-stimulation group. All participants underwent the Behavior Rating Inventory of Executive Function - Adult Version Scale (BRIEF-A) and Visual Analog Scales (VAS) measurements. Sixty-five healthy adults matched to the general condition of MUD patients were also recruited as healthy controls.

**Findings:** Patients with MUD had significantly worse executive function. iTBS groups had better treatment effects on the MUD group than the sham-stimulation group. Further Spearman rank correlation and stepwise multivariate regression analysis revealed that reduction rates of the total score of the BRIEF-A and subscale scores of the inhibition factor and working memory factor in the iTBS group positively correlated with improvements in craving. ROC curve analysis showed that working memory (AUC = 87.4%; 95% CI = 0.220, 0.631) and GEC (AUC = 0.761%; 95% CI = 0.209, 0.659) had predictive power to iTBS therapeutic efficacy. The cutoff values are 13.393 and 59.804, respectively.

**Conclusions:** The iTBS rTMS had a better therapeutic effect on the executive function of patients with MUD, and the improved executive function had the potential to become a predictor for the efficacy of iTBS modality for MUD treatment.

**Clinical Trial Registration:** ClinicalTrials.gov, identifier: ChiCTR2100046954.

## Introduction

Methamphetamine (METH) is an extremely dependent psychoactive substance with a high relapse rate for addiction, and the search for an effective treatment against it has been a challenge in the field of addiction medicine (1). Repetitive transcranial magnetic stimulation (rTMS) is a widely used physiotherapy technique for brain stimulation; several previous studies have found it to be effective against substance use disorder (SUD) (2–5). With the increasing emphasis on the problem of SUD, a large number of researchers have conducted several explorations on the modified aspects of rTMS treatment methods; for example, in recent clinical practice of addiction, a new rTMS modality, theta burst stimulation (TBS), has come into use (6, 7) TBS mainly consists of two modalities: intermittent theta burst stimulation (iTBS) and continuous theta burst stimulation (cTBS), which promote and inhibit excitation, respectively. The TBS modality is well tolerated and safe (8). The reports evaluating the stimulation modality of iTBS for treating depression have demonstrated its potential of being no less than high-frequency rTMS mode (9). In recent decades, iTBS has also been gradually used in the field of SUD. It was previously studied in patients with cocaine addiction where the amount of cocaine and frequency of its use were reduced after 30 sessions of iTBS treatment (10). In treating METH use disorder (MUD), iTBS stimulation was recently reported to reduce craving and improve cognition in MUD patients (7, 11, 12). Previous studies have shown that iTBS has a shorter duration of treatment (iTBS for 3–5 min vs. 10 Hz for 15 min), thus allowing treatment of more patients and reducing the rates of discomfort (13). However, the research on iTBS in the field of SUD treatment is still in its infancy.

Craving is a core characteristic of SUD and a major driver influencing relapse (14, 15). Craving is also effective in predicting the risk of relapse in SUD individuals and is often targeted for treating SUD. Also, it is critical to find predictors effective against craving, in-depth understand the mechanisms of craving, and further provide a basis for evaluating clinical therapeutics. Executive function, as an important part of cognitive function, has gained increasing attention in studies on SUD, which has found that long-term drug abuse would cause widespread impairment of executive function (16, 17). Lower executive function has been reported to be associated with relapse (18), and improving executive function reduces relapse and improves treatment outcomes for SUD (19). Executive function may therefore be an effective novel therapeutic target for improving drug addiction and relapse and a valid predictor of treatment efficacy for SUD.

This study employed iTBS rTMS modalities for treating MUD, explored the therapeutic efficacy of iTBS modes for craving and executive function in MUD patients, and further explored whether executive function can predict the therapeutic effect of rTMS on MUD.

## Methods and Materials

### Participants

**Figure 1:**
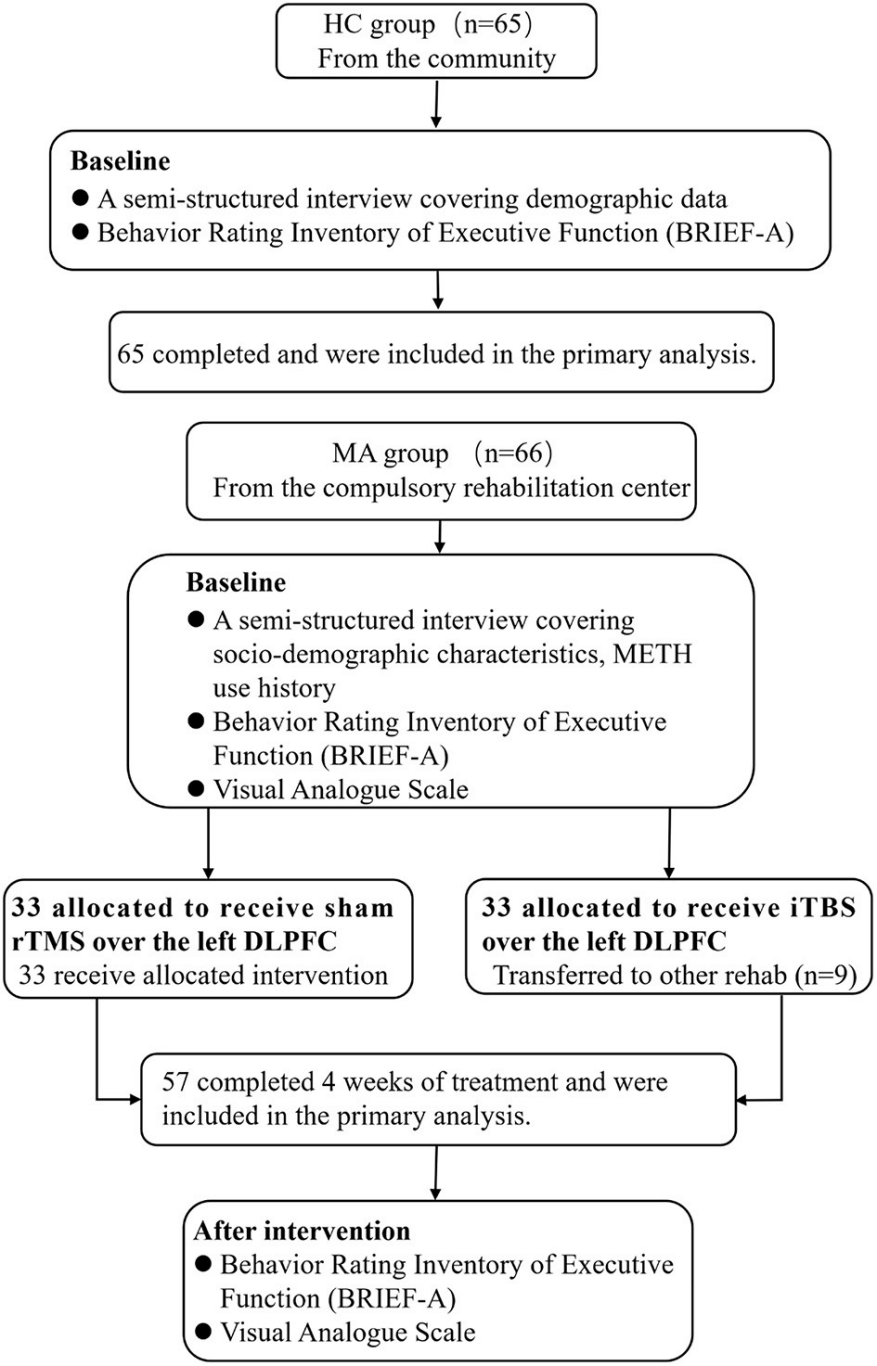
Flowchart of the study. HC, healthy control group; MUD, methamphetamine use disorder; iTBS, intermittent theta burst stimulation.

Sixty-six MUD subjects (MUD group; within 3 months of detoxification) from Bengbu Drug Rehabilitation Center met the Diagnostic and Statistical Manual of Mental Disorders (DSM-5) diagnostic criteria. The conformance to these diagnostic criteria was confirmed by an expert psychiatrist (associate professor). The study flowchart is shown in Figure 1.

The MUD group met the following inclusion criteria: (1) age 18–49 years; (2) normal eyesight and hearing; (3) received no other treatment during the experimental period; and (4) within 3 months of detoxification. The exclusion criteria for these subjects were as follows: (1) serious mental or neurological illness (e.g., schizophrenia, affective disorders, epilepsy, or Parkinson's disease); (2) dependence on substances other than nicotine (e.g., alcohol, heroin, and cocaine); and (3) any contraindications to rTMS. They were randomly assigned (using a computer-generated sequence of numbers) to the sham rTMS (*n* = 33) and iTBS groups (*n* = 33). Nine patients in the iTBS group who were transferred to other Drug Rehabilitation Centers for criminal reasons before study completion were not included in the dataset. Finally, there were 24 participants in the iTBS group. We did not find considerable differences in demographics and drug use history (e.g., age, years of education, age of onset, the numbers of relapse, METH use before abstinence, duration of METH use, baseline craving, and baseline executive function between finishers and non-finishers).

Individuals from a local community were included in the healthy control (HC) group (65 men). There was no difference between the HC and MUD groups in terms of demographics such as age, sex, marital status, and years of education. All the subjects volunteered to participate and signed an informed consent form for receiving rTMS treatment.

The Bengbu Medical College Institutional Review Board authorized this study (approval number: 2018-049). All experiments were performed in compliance with the regulatory approval.

### TMS Procedures

rTMS was administered by using the CCY-I TMS instrument (Yiruide Co., Wuhan, China). The figure-of-eight coil was applied in the iTBS protocol. The resting motor threshold (RMT) was identified by using the standardized procedure (20). At the beginning of each treatment session, the patient will be asked to wear a cloth EEG cap, and the position of intervention was determined by locating the positions of the F3 electrode for left DLPFC. Patients received 4 weeks of iTBS stimulation over the left DLPFC (5 days/week, 20 sessions). The iTBS parameters in this study were based on the previous safe parameters (11, 12). The iTBS group received 100% RMT 2 s on and 8 s off for 3 min with 600 pulses, three-pulse 50-Hz bursts every 200 ms (at 5 Hz). The sham rTMS group received the same parameters with the coil rotated 90° away from the skull. Patients in the sham group could hear the sound of the coil, but felt no real stimulation across the cortex. After treatment, patients were asked if they knew whether they received real or sham stimulation; all patients answered “real.” All participants were treated in separate rooms. All patients were assessed at every treatment session to record adverse events, including headache, insomnia, seizures, and dizziness.

### Blinding

Sixty-six patients who met the inclusion criteria were assigned to the two rTMS groups according to a computer-generated sequence of numbers. In this single-blinded study, all the participants were blinded to the truth or falsity of the stimulation received. One experimenter (who assessed the results) was also blinded to the acceptance of rTMS treatment received by the subjects.

### Assessments

Sixty-six MUD patients and 65 HCs were requested to complete a questionnaire, including demographics (age, education, etc.), and later complete the Behavior Rating Inventory of Executive Function - Adult Version Scale (BRIEF-A) in a separate, quiet room. Only MUD patients underwent a visual analog scale (VAS) evaluation, and details about their drug use history [age of onset (years), numbers of relapse, METH use before abstinence (g/every time), and duration of METH use (years)] were recorded.

Cue-induced craving: the VAS was used as a tool to determine the level of craving in MUD individuals. According to the methods reported in previous literature (21), we created a presentation consisting of 34 METH-related pictures, such as drug use sites, drug paraphernalia, drug powder, and drug use scenarios. Thirty-four pictures were presented each time for 5 min and played twice, and after stimulation the participants were asked to use a VAS to assess according to their own subjective feelings. The VAS is a straight line of about 10 cm in length, with 0 indicating no craving and 10 indicating extreme craving for METH. Cue-induced craving was used to allow participants to rate their current craving for the drug and was evaluated before and after TMS stimulation.

Chinese BRIEF-A was used to evaluate the executive function, which includes 75 items on nine subscales: inhibit, shift, emotional control, self-monitor, initiate, working memory, plan or organize, task monitor, and organization of materials. The first four constitute the Behavioral Regulation Index (BRI), the last five constitute the metacognitive index (MI), and the sum of the two index scores is the total score of the global executive composite (GEC). In this study, the internal consistency of Cronbach's α of this scale was 0.918, and the KMO test coefficient (Bartlett's test, *p* < 0.05) was 0.936, indicating that the scale had good reliability and validity.

Four weeks after rTMS treatment, 57 MUD patients were retested using the BRIEF-A and VAS.

### Statistical Analysis

Changes in craving = pre-experiment score of VAS – post-experiment score of VAS. The BRIEF-A reduction rate (RR) algorithm is listed as follows: when post-experiment score > pretreatment score, reduction rate = (pre-experiment score – post-experiment score)/(max – pre-experiment score) ×100%. When pre-experiment score = post-experiment score, reduction rate = 0; when post-experiment score < pre-experiment score, reduction rate = (pre-experiment score – post-experiment score)/(pre-experiment score – min) ×100%. “Max” and “min” indicate the upper and lower limits of the value range, respectively (22).

Data according with normal distribution were given as mean ±standard deviation (M±SD). Data according with skewness distribution were given as median (quartile spacing). Two-group differences were compared using the Student *t* test for continuous variables and the chi-square test for categorical variables, and the non-parametric Mann–Whitney *U* test for abnormally distributed variables. To investigate relationships between changes in craving (ΔCraving) and reduction rate in BRIEF-A, METH use history (age of onset, the number of relapse, METH use before abstinence, duration of METH), and demographics, Spearman correlation analysis was conducted in the MA group. A stepwise multivariate regression analysis of ΔCraving as a dependent variable was performed to investigate the effect of reduced rates of BRIEF-A and drug use history in the iTBS group. Receiver operating characteristic (ROC) curves were constructed at the most discriminating cutoff point values aiming at documenting the predictive power of ΔCraving for the iTBS therapeutic efficacy. The values of p were two-sided for all statistical tests. A value of *p* <0.05 was considered statistically significant. The SPSS program (version 25.0, SPSS, Chicago, IL, USA) was used for statistical analyses.

## Results

### Demographics, Drug Use History, and BRIEF-A Data in MUD and HC Groups

There were no differences between the MUD (*n* = 66) and HC groups (*n* = 65) in terms of years of education, age, and marital status (*p* > 0.05). The MUD group had significantly worse executive function than the HC group (*p* <0.05) in addition to initiate, plan or organize, task monitor, organization of materials, and MI (*p* > 0.05) (Table 1).

**Table 1:** Demographics, drug use history, and BRIEF-A data in MUD and healthy control groups.

	**MUD group**	**HC group**	***t/χ2***	***p***
	**(*n* =66)**	**(*n* = 65)**		
Age (years)	35.287 ± 6.243	34.154 ± 6.323	1.033	0.304
Years of education (years)	8.075 ± 3.416	7.554 ± 2.699	0.969	0.334
Married (%)	48.485	55.384	0.625	0.429
Age of onset (years)	26.485 ± 6.869			
The numbers of relapse	0.955 ± 0.867			
METH use before abstinence (g/ever time)	0.468 ± 0.320			
Duration of METH use (years)	8.803 ± 4.379			
**BRIEF-A**				
Inhibit	12.818 ± 3.229	10.892 ± 3.052	3.507	0.001
Shift	9.288 ± 2.332	8.200 ± 2.360	2.654	0.009
Emotional control	15.470 ± 3.900	13.554 ± 4.847	2.549	0.014
Self-monitor	9.364 ± 2.594	8.000 ± 2.250	3.212	0.002
Initiate	12.409 ± 2.893	11.646 ± 3.048	1.468	0.144
Working memory	12.258 ± 2.841	11.139 ± 2.936	2.217	0.028
Plan or organize	15.136 ± 3.867	13.908 ± 3.860	1.820	0.071
Task monitor	9.439 ± 2.240	9.385 ± 2.517	0.132	0.895
Organization of materials	11.849 ± 3.287	11.185 ± 3.716	1.083	0.281
BRI	46.394 ± 10.915	40.646 ± 11.393	3.239	0.002
MI	61.091 ± 13.528	57.262 ± 14.202	1.580	0.116
GEC	108.030 ± 23.626	97.908 ± 25.041	2.380	0.019

*Data according with normal distribution were given as mean ± standard deviation (M±SD). MUD, methamphetamine use disorder; HC, healthy control; BRIEF-A, Behavior Rating Inventory of Executive Function; BRI, Behavioral Regulation Index; MI, metacognitive index; GEC, global executive composite*.

### The Impairment of Executive Function Was Related to METH Use

Pearson correlation analysis was performed to determine the relationship between baseline BRIEF-A and METH use history in the MUD group (*n* = 66). The correlation analysis showed a significant and positive association with METH use before abstinence and baseline scores of BRIEF-A (inhibit, emotional control, self-monitor, initiate, plan or organize, BRI, MI, and GEC) (Table 2). Because the higher the BRIEF-A score is, the worse the executive function. The results of correlation analysis showed that the damage of executive function is positively correlated with the use of METH.

**Table 2:** Baseline executive dysfunction was mainly related to METH use before abstinence (*N* = 66).

**Baseline BRIEF-A**	**Age of onset (years)**	**The numbers of relapse**	**METH use before abstinence (g/ever time)**	**Duration of METH use(years)**
Inhibit	−0.206	0.024	0.304*	0.061
Shift	−0.022	0.144	0.113	−0.032
Emotional control	−0.109	0.111	0.273*	0.164
Self-monitor	−0.149	−0.020	0.290*	0.047
Initiate	0.034	−0.091	0.283*	−0.079
Working memory	−0.046	−0.008	0.170	0.146
Plan or organize	−0.008	−0.049	0.343**	<0.001
Task monitor	−0.144	0.105	0.135	0.123
Organization of materials	−0.009	−0.051	0.146	0.006
BRI	−0.140	0.073	0.280*	0.081
MI	−0.031	−0.030	0.252*	0.036
GEC	−0.082	0.017	0.274*	0.058

BRI, Behavioral Regulation Index; MI, metacognitive index; GEC, global executive composite. *p < 0.05, **p < 0.01.

### iTBS rTMS Treatments Exhibited Good Effects on MUD

Before the treatment, there were no differences between the sham and iTBS groups in terms of age, education, marital status, METH use history (age of onset, the number of relapse, METH use before abstinence, duration of METH), baseline executive function (inhibit, shift emotional control, self-monitor, initiate, working memory, plan or organize, task monitor, organization of materials, BRI, MI, GEC), and baseline craving (Table 3).

**Table 3:** Demographics, drug use history, BRIEF-A, and VAS scores in two MUD groups before treatment had no difference.

	**Sham group**	**iTBS group**	***t/χ2***	***p***
	**(*n* = 33)**	**(*n* = 24)**		
**Demographics**				
Age (years)	36.030 ± 6.626	34.545 ± 5.842	0.966	0.338
Years of education (years)	7.818 ± 3.770	8.333 ± 3.058	−0.610	0.544
Married (%)	57.575	39.393	2.184	0.139
**Drug use history**				
Age of onset (years)	27.545 ± 7.124	25.424 ± 6.538	1.260	0.212
The numbers of relapse	1.000(1.000)	1.000(0.500)	−0.864	0.387
METH use before abstinence (g/ever time)	0.481 ± 0.301	0.454 ± 0.341	0.341	0.735
Duration of METH use (years)	8.484 ± 4.528	9.121 ± 4.270	−0.587	0.559
**Baseline BRIEF-A**				
Inhibit	12.818 ± 3.504	12.818 ± 2.983	<0.001	1.000
Shift	8.848 ± 2.209	9.727 ± 2.401	−1.547	0.127
Emotional control	15.121 ± 3.747	15.818 ± 4.065	−0.724	0.472
Self-monitor	9.151 ± 2.762	9.575 ± 2.437	−0.662	0.511
Initiate	12.393 ± 3.009	12.454 ± 2.851	−0.042	0.966
Working memory	11.818 ± 2.822	12.697 ± 2.833	−1.262	0.211
Plan or organize	15.030 ± 4.171	15.242 ± 3.597	−0.221	0.826
Task monitor	9.151 ± 2.279	9.727 ± 2.267	−1.045	0.300
Organization of materials	12.060 ± 3.161	12.575 ± 3.345	−1.829	0.072
BRI	45.939 ± 10.985	47.939 ± 10.919	−0.742	0.461
MI	60.454 ± 13.809	62.697 ± 13.412	−0.946	0.348
GEC	106.393 ± 23.658	110.636 ± 23.832	−0.884	0.380
**Baseline craving**	2.848 ± 2.840	3.515 ± 3.571	−0.839	0.405

*Data according with normal distribution were given as mean ±standard deviation (M±SD). Data according with skewness distribution were given as median (quartile spacing). BRIEF-A, Behavior Rating Inventory of Executive Function. BRI, Behavioral Regulation Index; MI, metacognitive index; GEC, global executive composite*.

Comparisons of the reduction rate of BRIEF-A and changes of ΔCraving in the sham and iTBS groups after treatment were conducted. The Mann–Whitney U test showed that the iTBS group had an obvious improvement in ΔCraving and a reduction rate of inhibit, shift, emotional control, self-monitor, initiate, working memory, BRI, and GEC compared with the sham group (*p* < 0.05) (Table 4).

**Table 4:** Comparisons of reduction rate of BRIEF-A and changes of craving in sham group and iTBS group after treatment.

	**Sham group**	**iTBS group**	**Z**	**p**
	**(*n* = 33)**	**(*n* = 24)**		
**Changes of craving**				
ΔCraving	0(2.5)	1(3)	−2.112	0.045
**BRIEF-A (reductive rate, %)**				
Inhibit	−7.692(43.062)	38.750(74.107)	−3.528	<0.001
Shift	0(73.000)	50.000(100.000)	−2.378	0.017
Emotional control	−15.000(28.205)	41.428(72.916)	−4.019	<0.001
Self–monitor	0(44.446)	26.785(71.250)	−2.448	0.014
Initiate	−5.778(9.829)	−0.625(7.158)	−2.664	0.008
Working memory	0(53.109)	13.393(47.500)	−2.032	0.042
Plan or organize	0(68.611)	4.545(89.285)	−1.719	0.086
Task monitor	0(50.278)	17.143(66.250)	−1.946	0.052
Organization of materials	0(59.127)	5.555(68.750)	−1.143	0.253
BRI	−0.113(19.285)	33.772(60.162)	−3.969	<0.001
MI	9.093(56.681)	23.411(41.623)	−1.843	0.065
GEC	2.393(42.556)	30.308(45.166)	−3.071	0.002

*Data according with skewness distribution were given as median (quartile spacing). ΔCraving, changes of craving. BRI, Behavioral Regulation Index; MI, metacognitive index; GEC, global executive composite. Compared with the sham group*.

### Correlation Analyses of the Changes in Craving (ΔCraving) and Reduction Rate in BRIEF-A, METH Use History, and Demographics

**Figure 2:**
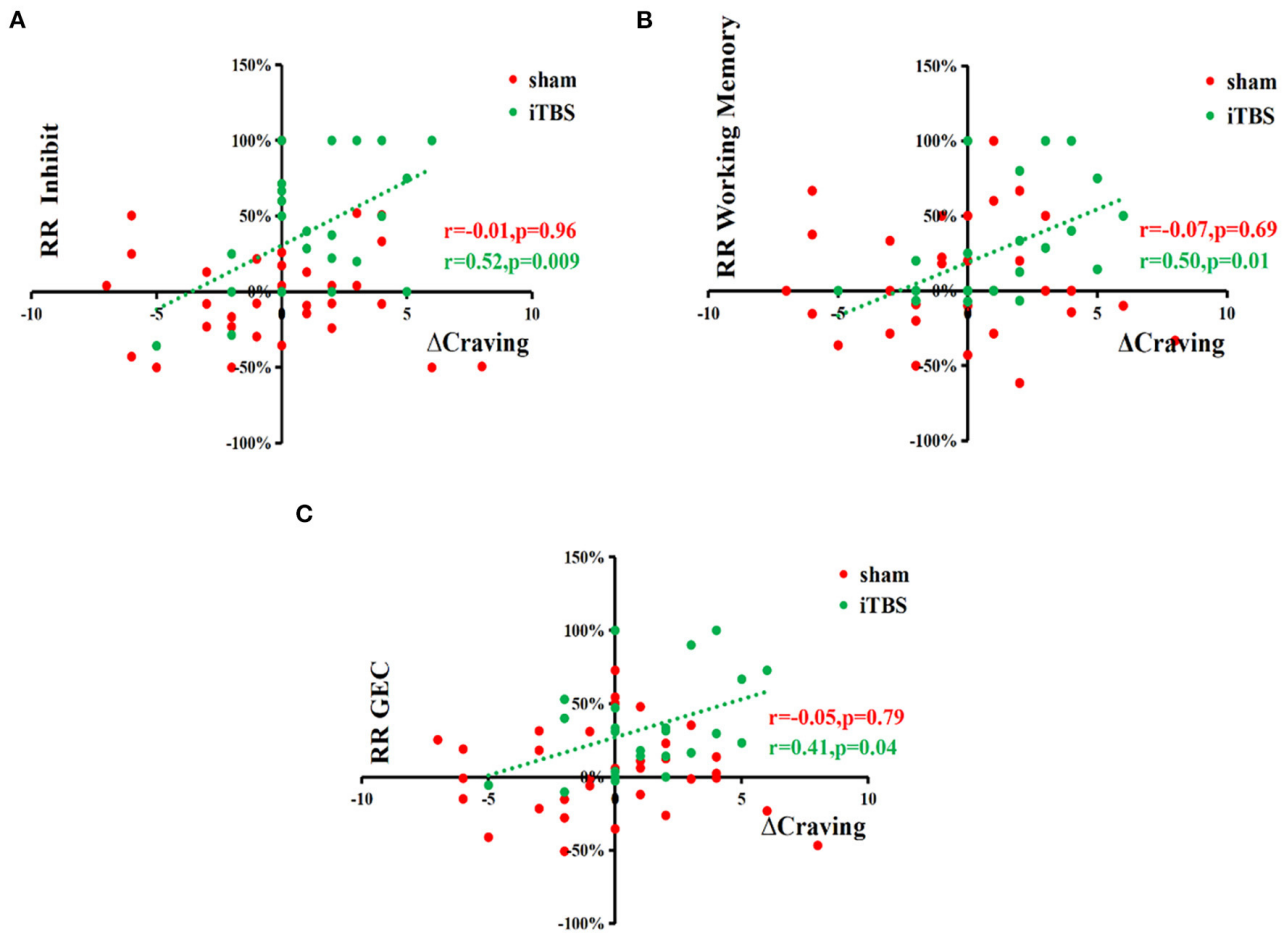
Correlation analyses show the positive relationship between changes in craving (ΔCraving) and reduced rates of subscale scores of inhibition, working memory, and GEC. Spearman rank correlation analysis between the changes of craving (ΔCraving) and reduction rate of BRIEF-A, METH use history data, and demographics, respectively, in two MUD groups showed a significantly and positively relationship between ΔCraving and reduction rate of inhibition and reduction rate of working Memory in the iTBS groups (*N* = 24). Other data showed no significant correlation. ΔCraving, changes of craving, RR, reduction rate.

Spearman rank correlation analysis showed a significant and positive relationship between changes in craving (ΔCraving) and reduced rates of subscale scores of inhibit (r = 0.44, *p* = 0.03), working memory (r = 0.58, *p* = 0.003), and GEC (r = 0.41, *p* = 0.04) in the iTBS group (*n* = 24). Other data showed no significant correlation (Figure 2).

### Stepwise Multivariate Regression Analysis of Changes in Craving (ΔCraving) as a Dependent Variable to Determine the Effect of Reduced Rate of BRIEF-A and Drug Use History

A stepwise multivariate regression analysis of ΔCraving as a dependent variable was performed to investigate the effect of reduced rates of BRIEF-A and drug use history in iTBS groups. Multivariate regression analysis showed that the reduction rates of GEC (β = 0.414, t = 2.134, *p* = 0.044) and subscale scores of inhibition (β = 0.523, *t* = 2.876, *p* = 0.009) and working memory (β = 0.500, *t* = 2.707, *p* = 0.013) were independently associated with ΔCraving in the iTBS group (*n* = 24) (Table 5). It indicated that improvement in inhibition, working memory subscale, and GEC could predicate the improvement in craving.

**Table 5:** Stepwise multivariate regression analysis with changes of craving (ΔCraving) as dependent variables to explore the effect of the reduction rate of BRIEF-A and drug use history.

**Group**	**Dependent variables**	**Independent variable**	**Unstandardized coefficients**		**Standardized coefficients**	**t**	***p***
			**β**	**SE**	**β**		
iTBS	ΔCraving	Reductive rate of inhibition	0.032	0.011	0.523	2.876	0.009
(*n* = 24)		Reductive rate of working memory	0.035	0.013	0.500	2.707	0.013
		Reductive rate of GEC	0.033	0.015	0.414	2.134	0.044

*Multivariate regression analysis showed that the reduction rates of GEC, inhibition, and working memory were independently associated with ΔCraving in the iTBS group. ΔCraving, changes of craving; GEC, global executive composite*.

### ROC Curve Analysis for Predictive Power of ΔCraving to iTBS Therapeutic Efficacy

**Figure 3:**
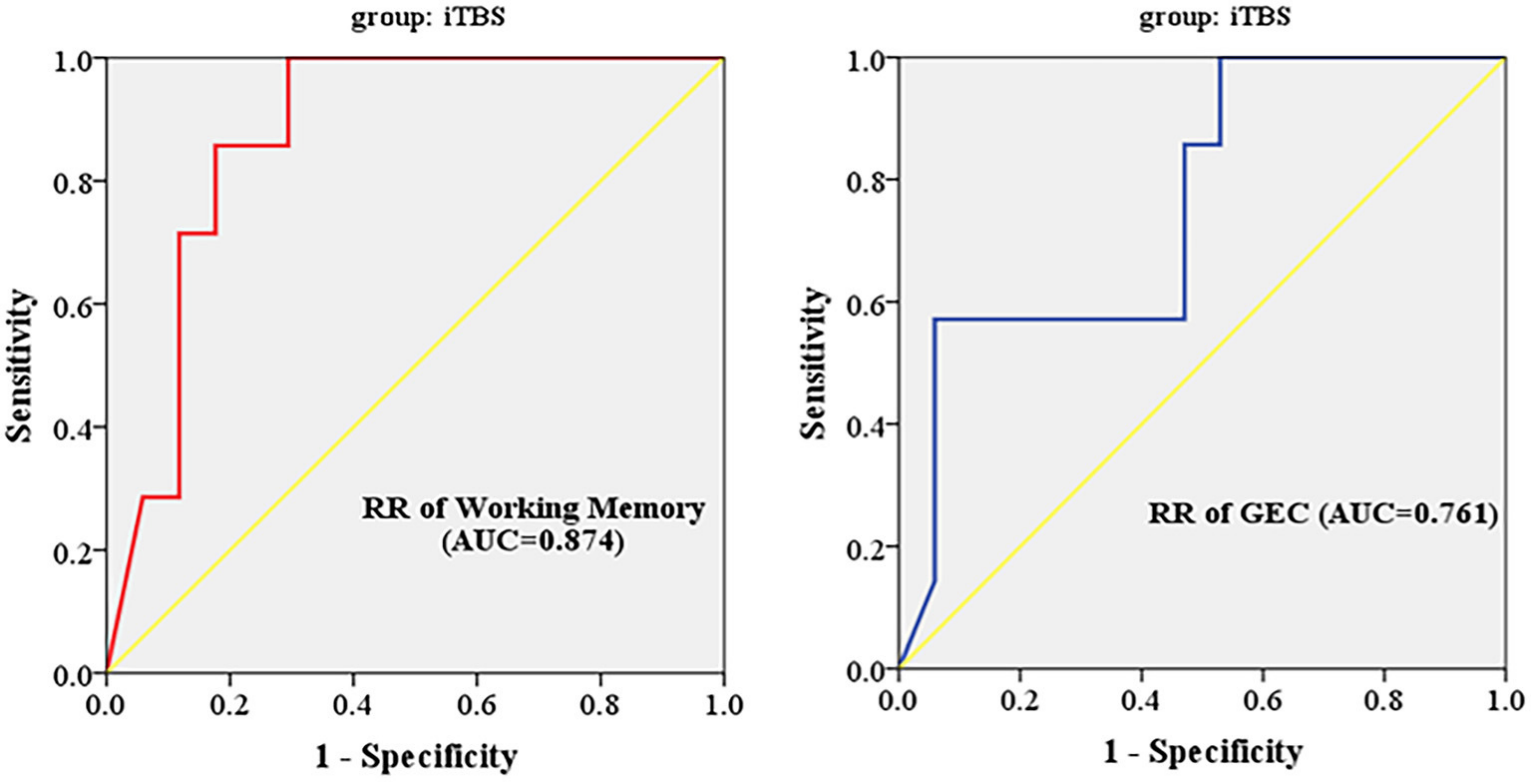
Working memory subscale scores and GEC had predictive power to iTBS therapeutic efficacy. RR, reduction rate; GEC, global executive composite; AUC, area under the curve; iTBS, intermittent theta burst stimulation.

ΔCraving ≤ 2 was regarded as ineffective treatment, and ΔCraving > 2 was regarded as effective treatment. The results of ROC curve analysis showed that inhibition subscale scores had no predictive power, and working memory subscale scores and GEC had predictive power to iTBS therapeutic efficacy. Finally, AUC, *p* value, 95% confidence interval (CI), sensitivity, specificity, and cutoff value are shown in Table 6. Working memory (AUC = 87.4%; 95% CI = 0.220, 0.631) had higher predictive power than GEC (AUC = 0.761%; 95% CI = 0.209, 0.659). The cutoff values are 13.393 and 59.804, respectively (Figure 3).

**Table 6:** ROC curve analysis for predictive power of reduction rates of inhibition, working memory, and GEC to iTBS therapeutic efficacy.

**Parameter**	**AUC**	***p***	**95% CI**	**Sensitivity (%)**	**Specificity (%)**	**Cutoff value**
Reduction rate of inhibition	0.710	0.112	0.294–0.871	57.1	88.2	0.453
Reduction rate of working memory	0.874	0.005	0.220–0.631	100	70.6	13.393
Reduction rate of GEC	0.761	0.049	0.209–0.659	57.1	94.1	59.804

*ROC curve analysis showed that inhibition subscale scores had no predictive power, and working memory subscale scores and GEC had a predictive power to iTBS therapeutic efficacy. AUC, area under the curve; CI, confidence interval; GEC, global executive composite*.

It indicated that improvement in executive function in the iTBS group might become a predictive factor for treatment efficacy in MUD patients.

## Discussion

### MUD Patients Exhibited Significant Impairment of Executive Function

Executive function, often viewed as a complex cognitive function primarily regulated by the prefrontal cortex, is a flexible set of cognitive abilities used by individuals in achieving a given goal. It includes a range of functions such as planning, working memory, control of impulses, inhibition, action generation, and monitoring (23). Impairment of executive function has an important role in the formation, maintenance, cessation, and relapse of SUD (24). Long-term substance abuse can lead to pervasive impairment of executive function, resulting in increased impulsive behavior and decreased self-control, in turn affecting the treatment effects of SUD and leading to an increased likelihood of relapse (25, 26).

Our study found that MUD patients exhibited significant impairment of executive function, which correlated with the dose of METH used. This result is consistent with previous research (16, 27).

### iTBS rTMS Treatments Exhibited Good Effects on MUD

Currently, researchers have conducted therapeutic explorations using rTMS for psychoneurological disorders and SUD. Previous studies have made continuous attempts for treatment modalities, from low frequency (28, 29) to high frequency (18, 30), and from rTMS to TBS or from the original stimulation site (e.g., L-DLPFC) to other sites (e.g., mPFC) (11). Current research suggested that the stimulation of the DLFPC region (area within the executive control network) by modalities such as high-frequency rTMS or iTBS effectively reduced craving, which might be associated with enhanced executive function (12, 31, 32).

Also, previous studies showed that using the iTBS parameter [three-pulse 50-Hz bursts at every 200 ms (i.e., at 5 Hz)], 2 s on and 8 s off for 5 min once per day, with 900 pulses in total, at 100% RMT, 5 days/week, 20 daily sessions, over the left DLPFC (12), or parameter [three-pulse 50-Hz bursts at every 200 ms (i.e. at 5 Hz)], 2 s on and 8 s off for 3 min once per day, with 600 pulses in total, at 70% RMT, twice-daily TBS over five consecutive days for a total of 10 sessions, over the left or right DLPFC (33), all reduced the craving scores of MUD patients compared with those before intervention and control groups.

We used the iTBS parameter [three-pulse 50-Hz bursts at every 200 ms (i.e., at 5 Hz), 2 s on and 8 s off for 3 min once per day, with 600 pulses in total, at 100% RMT, 5 days/week, 20 daily sessions, over the left DLPFC] to treat MUD patients, and the result showed that these modalities had an improved effect on impaired executive function and cue-induced craving. During this procedure, we did not observe seizures or other serious adverse reactions, and the safety of iTBS has been stated in previous reports (34, 35).

Executive function is primarily correlated with prefrontal lobe function; previous studies found that METH abuse caused prefrontal lobe damage, and prefrontal lobe damage was the biological basis by which METH caused impairment of executive function (16, 17, 36). Therefore, the therapeutic effect of iTBS on executive function can be achieved by activating DLPFC (major executive control network) and improving the function of the prefrontal lobe.

RTMS may improve executive function by increasing GABAergic levels in the prefrontal cortex (PFC). PFC is involved in executive function processes (37, 38). In PFC, the GABAergic interneurons maintain the normal functions of the PFC pyramidal cells through their inhibitory effects on the dopaminergic neurotransmitter (projecting from the VTA) and glutamatergic neurotransmitters (projecting from the thalamus) (39, 40). Therefore, reduced function of the GABA system can lead to executive dysfunction (41–43). Previous studies have found that the use of METH increases the glutamate level (44, 45) and dopamine level (46) in the PFC. Overactivation of the glutamatergic neurons and dopamine neurons by METH could produce excitatory neurotoxicity and cause cell apoptosis, finally resulting in executive dysfunction (47, 48). The PFC GABAergic interneurons suppress overactivation of the dopaminergic and glutamatergic neurons, and METH exacerbates these effects by disturbing the inhibitory function of the GABAergic interneurons (49, 50). More and more studies have found that theta-burst stimulation or 10-Hz rTMS can improve the level of GABA in PFC (51–53). It could be hypothesized that improving the function of the GABA system induced by rTMS would have a protective effect against METH-induced executive dysfunction.

### Improvement in Executive Function in the iTBS Group Might Become a Predictive Factor for Treatment Efficacy in MUD Patients

Previous research identified deficits in executive function and decision-making ability as significant predictors of relapse of SUD (19). This study found that the iTBS modality produced therapeutic efficacy for the executive function of MUD patients, which might predict the therapeutic efficacy of iTBS for METH addiction.

The theta rhythm parameters for the iTBS modality are developed according to the physiological functions of rodents and the human brain, and theta rhythm was similar to the physiological rhythm of the human cerebral cortex compared with high-frequency stimulation. Theta rhythm was found in multiple cerebral regions and was related to multiple cognitive functions, e.g., working memory, episodic memory, and executive function (54, 55). Normal theta rhythm oscillation is considered a predictor of good performance in executive function (56). Human brain imaging studies have shown enhanced coupling of theta oscillations between the hippocampus and DLPFC during cognitive activity (57). Disrupted coupling of theta oscillations between the hippocampus and DLPFC occurs in patients with psychiatric disorders in which cognitive impairment is the predominant feature (58). Clinical and animal experiments have also shown that substance abuse causes abnormal theta oscillations in multiple brain regions (59–61). Thus, iTBS patterns that fit the endogenous theta rhythm pattern may be favorable for modulating executive function.

rTMS can modulate cortical excitability, neuronal plasticity, and brain functional connectivity (62, 63). The magnitude of motor-evoked potentials elicited by the iTBS modality was stronger than other stimulation modalities (64). The increased number of pulses of iTBS stimulation had dose-dependent effects on the resulting cortical excitability and functional connectivity (65). The strong cortical excitability produced by iTBS might lead to a good therapeutic effect of executive function.

### Limitations

(1) Because of regional limitations, we only examined male MUD patients; further studies required that both male and female MUD patients be included.
(2) A sole executive function scale was used in this study. In future studies, a battery of neurocognitive function tests will be employed to explore the predictive effects of executive function on MUD treatment.
(3) In this study, we have tried to decrease the potential deviation of the single blinding method and improve the therapeutic comfort. In addition, all the subjects were informed that they would not be allowed to reveal any treatment details and not to discuss the details of treatment with each other. However, there is still a common problem in the rTMS clinical trial; that is, the slight irritant pain and discomfort of treatment may weaken the blinding method. In the future, it is significant to adopt more comprehensive blinding methods and increase the sample size.
(4) This study assessed the short-term predictive role of executive function. Further follow-up is needed to observe the long-term predictive effect of executive function on the chronic and repeating feature of MUD.

In summary, this study found that MUD could cause impairment of executive function. iTBS rTMS modality produced therapeutic efficacy for craving and executive function of MUD patients. The reduction rates of total score of the BRIEF-A and subscale scores of the working memory factor might be a predictor for the therapeutic efficacy of iTBS. In addition, this study also provides new ideas for the treatment of MUD by improving executive function.

## Data Availability Statement

The original contributions presented in the study are included in the article/supplementary material, further inquiries can be directed to the corresponding author/s.

## Ethics Statement

The studies involving human participants were reviewed and approved by Compulsory Isolated Drug Rehabilitation Center, Bengbu, Anhui, China. The patients/participants provided their written informed consent to participate in this study.

## Author Contributions

L-JW, L-LM, and D-LJ: conceptualization and methodology. YW, Y-DZ, and XS: data curation. D-LJ, H-SX, L-JW, and Z-XR: formal analysis. D-LJ, L-JW, Z-XR, and W-JW: funding acquisition. H-JT, QL, W-JW, P-PS, LZhu, TW, Y-JW, HG, LZha, H-FW, L-XC, TW, YW, L-YL, and WZ: investigation. D-LJ and H-SX: project administration, supervision, and validation. R-DZ and LZha: resource and software. D-LJ, H-SX, and Z-XR: visualization. H-SX and Z-XR: writing—original draft. D-LJ and Z-XR: writing—review and editing. All authors contributed to the article and approved the submitted version.

## Funding

This project was supported by the Provincial Natural Science Foundation of Anhui (1908085MH278), Shanghai Key Laboratory of Psychotic Disorders Open Grant (13dz2260500), Program of Bengbu Medical College Science and Technology Development (2020byzd021, BYKF1820, BYKF1818, 2020byzd022), Anhui Provincial Education Department Humanities and Social Science Key Project (SK2019A0181), Bengbu Medical College Innovative Training Program for Postgraduate Students (Byycx20048, Byycx20008, Byycx21025, Byycx21037, Byycxz21039), Innovative Training Program for Chinese College Students (202010367024), Natural Science Research Project of Anhui Educational Committee (KJ2018A1017), Bengbu City - Bengbu Medical College Joint Science and Technology Project (BYLK201822), and Bengbu Medical College Key Laboratory of Addiction Medicine (29-3). All funders did not interfere in the study design, collection, analysis, interpretation, or writing of manuscript.

## Conflict of Interest

The authors declare that the research was conducted in the absence of any commercial or financial relationships that could be construed as a potential conflict of interest.

## Publisher's Note

All claims expressed in this article are solely those of the authors and do not necessarily represent those of their affiliated organizations, or those of the publisher, the editors and the reviewers. Any product that may be evaluated in this article, or claim that may be made by its manufacturer, is not guaranteed or endorsed by the publisher.
